# Development of a rapid phage-based method for the detection of viable *Mycobacterium avium* subsp. *paratuberculosis* in blood within 48 h^☆^

**DOI:** 10.1016/j.mimet.2013.06.015

**Published:** 2013-09

**Authors:** Benjamin M.C. Swift, Emily J. Denton, Sophie A. Mahendran, Jonathan N. Huxley, Catherine E.D. Rees

**Affiliations:** a School of Biosciences, University of Nottingham, Sutton Bonington Campus, Loughborough, Leics, LE12 5RD, UK; b School of Veterinary and Medicine Science, University of Nottingham, Sutton Bonington Campus, Loughborough, Leics, LE12 5RD, UK

**Keywords:** PMMS, Peptide mediated magnetic separation, FPTB, *FASTplaqueTB* assay, MP, Media Plus, Bacteriophage, Johne's disease, Magnetic separation, Paratuberculosis, Rapid detection

## Abstract

The aim of this study was to develop a methodology to rapidly detect viable *Mycobacterium avium* subsp. *paratuberculosis* (MAP) in clinical blood samples. MAP cells spiked into commercially available blood were recovered using optimised peptide-mediated magnetic separation (PMMS) and detected using a phage-based method, and the identity of the cells detected confirmed using nested-PCR amplification of MAP signature sequences (IS*900*). The limit of detection was determined to be 10 MAP cells per ml of blood and was used to detect MAP present in clinical bovine blood samples. Using the PMMS-phage method there was no difference when detecting MAP from whole blood or from isolated buffy coat. MAP was detected in animals that were milk-ELISA positive (15 animals) by PMMS-phage and no MAP was detected in blood samples from an accredited Johne's disease free herd (5 animals). In a set of samples from one herd (10 animals) that came from animals with variable milk ELISA status, the PMMS-phage results agreed with the positive milk-ELISA results in all but one case. These results show that the PMMS-phage method can detect MAP present in naturally infected blood. Total assay time is 48 h and, unlike PCR-based detection tests, only viable cells are detected. A rapid method for detecting MAP in blood could further the understanding of disseminated infection in animals with Johne's disease.

## Highlights

• Optimisation of efficient MAP cell capture in blood using magnetic separation
• Found a limit of detection of 10^1^ pfu ml^− 1^ in spiked blood
• Optimised a PCR to detect signature MAP DNA sequences from just one plaque
• We successfully detected viable MAP in naturally infected animals within 48 h

## 1 Introduction

*Mycobacterium avium* subsp*. paratuberculosis* (MAP) is the causative agent of Johne's disease which is a wasting disease of cattle and other ruminants that results in lower meat and milk yields, and significant financial losses to both the dairy and beef industries (Raizman et al., 2009). Paratuberculosis occurs throughout the world and is considered endemic in many countries (Fridriksdottir et al., 1999). MAP is transmitted vertically through contaminated milk and colostrum or horizontally via contaminated feed (Whittington and Sergeant, 2001). The disease can be controlled but early detection and reliable diagnostics are paramount in stopping transmission between animals. In Europe herd prevalence ranges from 7% to 55% but the subclinical nature of the disease and the limitations of available diagnostic tests can result in underreporting (Manning and Collins, 2001).

The identification of MAP in the blood of animals susceptible to Johne's disease has been carried out using techniques such as PCR and culture (see Bower et al., 2010 for a review). The use of PCR, although rapid does not distinguish between live and dead organisms. Culture is extremely slow and can take up to 16 weeks to form colonies and in some cases requires decontamination, which can reduce the number of viable cells in a sample (Grant et al., 2003; Gumber and Whittington, 2007). Although Bower et al. (2010) has indicated that some decontamination methods may affect certain MAP strains more than others. Most recently Bower et al. (2010) has described an optimised method for culture of MAP from decontaminated erythrocyte-lysed washed buffy coat, with a reported sensitivity of 10 MAP per ml of spiked whole blood, but this still required up to 12 weeks incubation. Hence this long time required for culture can hamper efforts to understand different phases of disease and determining when an animal develops disseminated infection.

The *FASTplaqueTB™* assay (FPTB; Lab21, UK) is a phage-based detection method for human tuberculosis that has been adapted for the detection of viable MAP in milk and cheese (Altic et al., 2007; Botsaris et al., 2010; Stanley et al., 2007). To increase the specificity of the method the phage-based assay was combined with PCR amplification of the multi-copy IS*900* element. During the development of the phage-based assay it was noted that substances present in the sample matrix can inhibit phage infection. Sample processing was required to ensure these are removed. Magnetic separation is a very simple method of capturing and concentrating cells from a matrix using magnet beads coated with a specific binding agent (either antibody or peptide). MAP-specific binding peptides coupled to magnetic beads described by Stratmann et al. (2006), have been used to recover MAP cells from milk samples (Foddai et al., 2010).

The aim of this study was to combine existing technologies to develop a novel protocol that could be used to rapidly detect MAP cells in blood using a combined phage-PCR approach, and then to test whether this could be used to detect viable MAP in clinical blood samples.

## 2 Materials and methods

### 2.1 Bacterial strains, bacteriophage and growth media

MAP strains K10 and ATCC 19698 were used in the initial experiments to optimise the phage assay. The *Mycobacterium smegmatis* strain and the bacteriophage used (Actiphage) were those supplied in the *FASTPlaqueTB™* kit. All cultures of MAP were prepared using *FASTPlaqueTB™* Media Plus (modified 7H9/OADC media) supplemented with Mycobactin J (2 μg μl^− 1^; Synbiotics Corporation, France).

To culture from beads, 100 μl of each sample, after PMMS (Section 2.3) were inoculated on Herrold's egg yolk medium (HEYM) slopes supplemented with Mycobactin J (BD, France). After inoculation, tubes were incubated slanted with the caps lightly screwed at 37 °C. After one week of inoculation slopes were examined for growth of contaminating organisms. After two weeks the caps were sealed and the slopes incubated up right. The slopes were then examined every week for 16 weeks, and every month up to six months after for growth. After six months, if there was no visible growth, a slope wash detection method was carried out.

### 2.2 Slope wash detection of MAP growth

The slope wash was based on a method by Williams and Monif (2009). Briefly, 1 ml of sterile reverse osmosis (RO) water was added to a slope with no visible growth. Using a sterile loop, the surface of the agar was gently scraped to loosen any cells present. The slopes were vortexed for 30 s and the liquid transferred to 1.5 ml centrifuge tube and centrifuged (15 min, 17000 ×*g*). The supernatant was removed, frozen at − 80 °C and then rapidly thawed before boiling for 10 min to induce cell lysis. The samples were then centrifuged for 3 min (17000 ×*g*) and the supernatant (10 μl) used as template DNA for PCR amplification of IS*900* (Section 2.6).

### 2.3 Preparation of peptide-coated magnetic beads

Biotinylated peptides were supplied by Cambridge Peptides Ltd. Magnetic beads (Dynabeads - MyOne Tosylactivated, Invitrogen) were individually coated with peptides aMp3 and aMptD (Stratmann et al., 2006) according to the manufacturers' instructions.

### 2.4 Sample preparation and recovery of MAP cells from blood

For development of the assay, MAP cells were added to commercial horse or sheep's blood (Oxoid, UK). To recover MAP cells, 1 ml blood samples were diluted with 9 ml of Media Plus. Peptide mediated magnetic separation was performed using an adaptation of the method described (Foddai et al., 2010) by adding 10 μl of peptide coated beads (5 μl each of aMp3- and aMptD-coated beads) to each sample and mixing at 18 rpm for 30 min (Dynabeads-MX mixer, Invitrogen). Beads and bound MAP cells were recovered by centrifugation at 4500 ×*g* for 15 min. The supernatant was removed and the beads washed using 9 ml of fresh Media Plus. The beads were again recovered by centrifugation (4500 ×*g*, 15 min) and finally resuspended in 1 ml of Media Plus before being transferred into a microcentrifuge tube for PMMS. Finally, the beads resuspended in 1 ml of Media Plus and Mycobacteria cells present detected using the FPTB assay reagents.

### 2.5 Detection and enumeration of MAP using the FASTPlaqueTB™ (FPTB) assay

The FPTB (Lab21, Cambridge, UK) assay was carried out according to the manufacturer's instructions. To perform the assay, samples containing MAP are mixed with a broad spectrum mycobacteriophage; D29. After the infection period any extracellular phage are inactivated using a virucide; only phage that have successfully infected a cell are protected from the virucide. The virucide is then neutralised by dilution and infected cells are then plated in a lawn of fast growing *M. smegmatis* using soft agar. Lysis of the infected cells releases new phage which then infect the *M. smegmatis* cells and leads to the formation of a plaque in the lawn. Hence each plaque formed represents one MAP cell in the original sample. Standard FPTB assay positive and negative controls were used each time the assay was performed. Enumeration of MAP cells in inocula was determined using the modification of the FPTB assay as described by Rees and Botsaris (2012) which involves diluting samples until countable numbers of plaques are obtained (data reported as pfu ml^− 1^).

### 2.6 PCR for the identification of MAP cells

When a MAP cell is present in the initial sample, its DNA is preserved in the centre of the plaque (Stanley et al., 2007). Identification of the cell detected within single plaques was achieved by PCR amplification of IS*900* signature sequences from this DNA using a modification of the plaque-PCR method described by (Botsaris et al., 2010). In this study DNA was extracted from five plaques and concentrated from plaque agar using Zymo-Gel DNA Recovery Spin Columns™, (ZymoResearch, USA). IS*900*-specific nested PCR (Bull et al., 2003) was performed using a 5 μl sample of DNA as template. Purified MAP K10 DNA was used as a positive PCR control and DNA extracted from plaques only containing *M. smegmatis* cells (the FPTB positive control samples) as a negative control.

### 2.7 Detection of MAP in clinical blood samples

Blood samples were provided as superfluous material under the Veterinary Surgeons Act as part of an on-going herd health screening programme. The study protocol was approved by the University of Nottingham, School of Veterinary Medicine and Science ethical review panel prior to sample usage. Blood samples were collected from nine cows, which had produced positive Johne's milk ELISA test results on three separate occasions (Set A). Blood samples were collected from five cows that belong to an accredited Johne's disease-free herd (Set B). Before sampling, the site of venipuncture was cleaned twice with alcohol. Blood was drawn into either sterile sodium heparin Vacutainer tubes (for phage assay) or plain Vacutainer tubes for blood ELISA. The blood ELISA was performed by a commercial laboratory (Nationwide Laboratories, Leeds, UK).

### 2.8 Isolation of buffy coat and plasma

The isolation of the buffy coat from cows blood was carried out using Ficoll-Paque Plus (GE Healthcare Life Sciences, UK). The buffy coat layer from 2 ml of whole blood was isolated according to the manufacturer's instructions. The plasma layer was also taken and resuspended after the final centrifugation in MP. The samples were then processed as whole blood in Section 2.4.

## 3 Results

### 3.1 Optimisation of PMMS and sample preparation

**Fig. 1:**
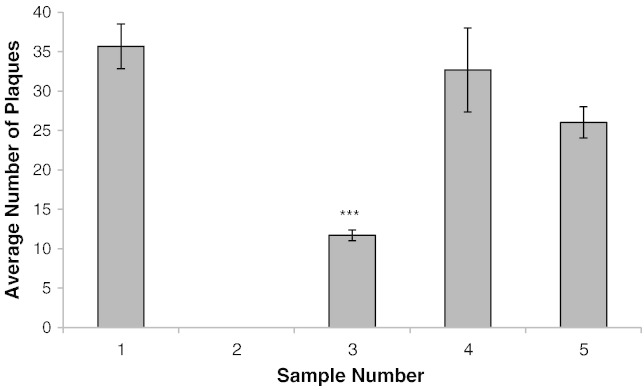
**Effect of blood on detection of MAP by PMMS-phage assay.** Graph showing plaque numbers recovered after performing the PMMS and phage assay on: Sample 1; MAP in 1 ml Media Plus. Sample 2; MAP in 1 ml of horse blood. Sample 3; MAP in 1 ml of sheep blood. Sample 4; MAP in 1 ml of sheep blood diluted 1:10 Media Plus. Sample 5; MAP in 1 ml of sheep blood diluted 1:50 Media Plus. A One-way ANOVA, followed by the Dunnett's test was used to analyse significance (*P < 0.001) in the reduction of plaque number when compared to Sample 1. Error bars represent the standard deviations of the means of number of plaques recovered from the phage assay performed in triplicate.

To develop the method, MAP cells were spiked into commercially available blood. To determine the efficiency of the PMMS recovery, 3.5 × 10^1^ pfu.ml^− 1^ was spiked into blood. Magnetic recovery of beads directly from blood samples was found to be inefficient (over 90% loss of sample). Therefore magnetic recovery was replaced by centrifugation which improved bead capture. Using this method, when MAP was spiked into horse blood, still no cells were detectable. However when sheep blood was used, 33% of the cells were recovered (Fig. 1). Assuming that the blood was inhibiting either the peptide binding or phage assay, the blood was diluted using FPTB Media Plus. After dilution the number of MAP cells detected from samples was significantly higher (*P* < 0.01) than that recovered from the undiluted blood, resulting in 92% recovery of MAP for a 1 in 10 dilution and 73% when a 1 in 50 dilution of the sample was used (Fig. 1). Accordingly, a 1 in 10 dilution was adopted as the standard method as it resulted in the most efficient recovery of MAP cells.

### 3.2 Determining limit of detection of PMMS-phage method

The number of MAP cells in a liquid culture was first determined using the modified FPTB assay (Section 2.5). These cultures were then diluted and spiked into sheep blood at different levels, down to approximately 1 MAP cell per ml. Using these samples, it was found that the optimised PMMS-phage method was able to reproducibly detect 10 MAP cells per ml of blood (Table 1).

### 3.3 Optimisation of nested-IS900 plaque-PCR

**Fig. 2:**
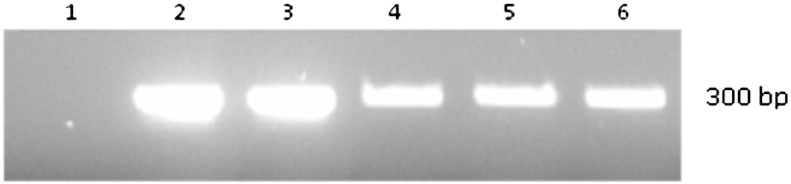
**Confirming detection of MAP DNA from mixed plaque samples.** Nested-PCR amplification of the 300 bp IS*900* DNA region specific for MAP (Bull et al., 2003). Lane 1; DNA extracted from 5 *M. smegmatis* plaques. Lanes 2; DNA extracted from 5 MAP plaques. Lane 3; 4 MAP plaques mixed with 1 *M. smegmatis* plaque. Lane 4; 3 MAP plaques mixed with 2 *M. smegmatis* plaques. Lane 5; 2 MAP plaques mixed with 3 *M. smegmatis* plaques. Lane 6; 1 MAP plaque mixed with 4 *M. smegmatis* plaques.

To confirm the detection of MAP by the phage assay, Stanley et al. (2007) extracted DNA from individual plaques and carried out a MAP specific PCR. In this study DNA was extracted from 5 plaques using a gel extraction kit and a nested-PCR (Bull et al., 2003) was used to amplify IS*900* signature sequences. Using this approach IS*900* DNA was always amplified from DNA extracted from the five plaques. To confirm that it was still possible to detect the DNA from a single MAP plaque within this sample, agar extracted from one MAP-positive and four MAP-negative plaques were mixed together. Even at this low concentration of target DNA, IS*900* DNA was routinely detectable (Fig. 2).

### 3.4 Detection of MAP in clinical blood samples

To determine whether the test developed in the laboratory was applicable to clinical blood samples, the optimised method was used to test bovine blood samples. Samples from a farm with a known Johne's disease problem were obtained from nine animals that had given three positive milk-ELISA test results (Set A). In addition five samples were obtained from an accredited Johne's disease-free herd (Set B). For comparative purposes, blood ELISA assays were performed in parallel with the PMMS-phage method. All of the animals in Set B gave negative blood ELISA results whereas all animals in Set A gave a positive blood ELISA result, except cow 8 (Table 2).

The results from the PMMS-phage method showed viable MAP cells were detected in all nine of the samples from Set A and no MAP was detected in any samples from Set B (Table 2). Two of the samples from Set B produced plaques (animal 11 and 13), but the IS*900* PCR did not detect any MAP DNA in these plaques indicating that no MAP cells were detected.

### 3.5 Comparison of MAP detection from whole blood and the buffy coat

To determine whether the number of MAP cells detected could be improved by isolation of the buffy coat layer, blood samples were obtained from a second set of ten animals, which now included cows that have given strong, intermediate or negative milk ELISA test results at the last time of testing (Set C). Blood ELISA tests were again performed, and antigens against MAP were detected in 4 out of the 10 animals (Table 3).

Each blood sample was tested using the PMMS-phage method both using whole blood and after buffy coat preparation. PCR-positive MAP plaques were detected in eight of the ten blood samples irrespective of the method of sample preparation, and there was no significant difference (*P >* 0.05) between the number of plaques isolated from whole blood or from the buffy coat layer (Table 3). After buffy coat isolation, the plasma fraction was recovered, but MAP was not detected using the PMMS-phage method in these samples (data not shown).

### 3.6 Culture of MAP following PMMS of blood

For the blood samples from Set C, culture was performed after PMMS of both whole blood and buffy coat layers using 0.1 ml samples plated onto Mycobactin J HEYM slopes. No chemical decontamination was performed, and no loss of samples to contamination was seen. However no growth was seen in any of the samples and the absence of any detectable MAP growth was confirmed using slope-wash and direct IS*900* PCR.

### 3.7 Reproducibility of the PMMS-phage method

To give an indication of the reproducibility, the phage assay was repeated for all blood samples twice, independently. There was a good agreement (r^2^ = 0.73) between the two independent test results for each blood sample tested. The results gained for the MAP-positive samples (Sets A and C) ranged from 3 to 35 pfu ml^− 1^, indicating that only low numbers of cells were detected. For MAP-negative samples (defined as plaques that were IS900-PCR negative) the number of plaques was 5 or below (Tables 2 and 3).

## 4 Discussion

The use of PMMS to recover cells from the sample has two benefits; it allows concentration of cells and does not affect the viability of MAP. The main obstacle for development of the assay was to achieve efficient capture of MAP cells in blood samples. Diluting the blood sample 1 in 10 using modified Media Plus (modified 7H9 media) gave the best improvement in recovery of MAP cells from spiked blood samples. The viscosity of horse blood is much higher than that of sheep blood (Windberger et al., 2003) and therefore limitation of bead movement in the sample may have hindered capture of MAP cells. Interestingly, the addition of Media Plus to the blood samples induced lysis of the blood cells. This may have contributed to the success of the assay when using clinical blood samples, since any intracellular MAP cells would be released into the medium and available for both PMMS and phage infection. This may also explain why no difference was seen in the efficiency of MAP detection in whole blood compared to isolated buffy coat layer. Thus negating the need to prepare the buffy coat layer before testing blood samples using this phage detection method.

The cows from Set A were selected using standard current diagnostic criteria for Johne's disease which requires repeat testing over a period of six months. The blood ELISA results for these animals agreed with this diagnosis in all but one case (cow #8) which gave an indeterminate test result. In contrast the PMMS-phage method detected viable organisms in blood samples from all animals in Set A, and this result agreed with the milk ELISA results. In Set B, two samples (animals 11 and 13) produced MAP DNA negative plaques, which may be because the virucide did not destroy all the phage before plating in the lawn of *M. smegmatis*. Break-through has been reported before when performing the FPTB assay but the introduction of the PCR identification step overcomes this problem. Hence in this study samples are not scored as MAP-positive unless the IS*900* sequence can be amplified from the plaque.

In Set C, three of the animals gave negative milk and blood ELISA test results and two of these were also negative for MAP using the PMMS-phage method. However the PMMS-phage method detected viable MAP in the blood from one animal (cow #22), and the plaque numbers were equivalent to those of the majority of the other milk ELISA-positive animals. This indicates that results gained using a method that directly detects the viable organism can differ from test results based on the immune response of the animal to infection. Further work is now needed to understand the relationship between the immune response of the animal and the presence of viable organisms in the blood.

When PMMS-phage MAP-positive samples were cultured, no growth was detected. Since the plaque number indicates that there were fewer than 42 MAP cells per ml in all of these samples, the lack of growth is to be expected as this number is below the limit of detection for the culture method used. MAP has been detected in blood samples by PCR-based methods and culture (Bower et al., 2010, 2011; Gwozdz et al., 2000; Naser et al., 2004). Although viable cells can be cultured from these samples, the time required makes this of limited practical value and the need for decontamination before culture may reduce the number of viable cells present in a sample (Reddacliff et al., 2003). Interestingly here, following PMMS of blood samples, no contamination of cultures was seen suggesting that the selectivity of the PMMS followed by the extensive washing used in this method is sufficient to remove contaminating microflora from the sample without the need to apply chemical decontamination.

In contrast to culture, PCR-based MAP detection methods are more rapid but they do not determine the viability of the cell detected. The results gained here show that the combined PMMS-phage-PCR assay can achieve rapid detection and identification of viable MAP in clinical samples within 48 h, and in addition provides an indication of the number of cells present in the sample. The limit of detection determined here is in agreement with an optimised MAP blood culture method reported by Bower et al (2010) but this method requires a 12 week incubation before results are available.

## 5 Conclusion

Here we have shown that we are able to detect viable MAP cells from spiked blood samples within 48 h, with a limit of detection of 10^1^ cells per millilitre of blood which can be combined with a sensitive PCR assay that specifically identifies the organism detected. The method was also tested using clinical blood samples, by selecting animals that were presumptively identified as having Johne's disease on the basis of repeated milk ELISA test results. Although this study was limited to small sample set, this is the first demonstration of a new method that can directly detect viable organisms in blood. This assay will allow researchers to better understand the development of disseminated infection, and may therefore lead to a better understanding of the pathogenesis of the disease and the immune response of the animal (Bower et al., 2010). In addition we believe that the assay could also be applied to blood samples from all susceptible animal species.

## Figures and Tables

**Table 1:** Limit of detection of phage assay in spiked sheep blood.

Approx. number of MAP cells in inoculum (pfu)	Number of MAP detected (pfu)
10^4^	Confluent a
10^3^	TNTC b
10^2^	151
10^1^	9
10^0^	0

a Confluent: lysis of 80% to 90% of the lawn of *M. smegmatis* cells.

b TNTC: Too numerous to count; merging of plaques.

**Table 2:** Results of analysis of blood samples from animals in Sets A and B.

Cow Number		Milk ELISA Status (3 tests)	Blood ELISA Status (OD Readings)		Plaque Number a		IS*900* Plaque PCR
Set A	1	+	+	(190)	35	27	+
	2	+	+	(> 227)	15	13	+
	3	+	+	(221)	19	25	+
	4	+	+	(111)	31	31	+
	5	+	+	(> 227)	11	25	+
	6	+	+	(> 227)	10	10	+
	7	+	+	(> 227)	35	29	+
	8	+	−	(1.47)	10	18	+
	9	+	+	(193)	5	9	+
Set B	10	−	−		0	0	NR
	11	−	−		2	0	−
	12	−	−		0	0	NR
	13	−	−		1	0	−
	14	−	−		0	0	NR

Numbers 1–9 represent Set A, Numbers 10–19 represent Set B and Numbers 10–14 represent set B. NR - ‘not required’ shows there were no plaques formed, therefore no PCR required.

a – Values show the numbers of plaques obtained in two independently tested samples.

**Table 3:** Results of analysis of blood samples from Set C animals.

Cow Number	Milk ELISA Status (most recent a)	Blood ELISA Status	Plaque Number				Plaque PCR	Culture
			Whole Blood b		Buffy Coat b			
**15**	Red	−	20	25	38	42	+	−
**16**	Red	+	3	7	22	21	+	−
**17**	Red	+	22	15	28	32	+	−
**18**	Red	+	12	3	17	12	+	−
**19**	Red	−	13	23	15	5	+	−
**20**	Red	+	8	6	9	5	+	−
**21**	Amber	−	21	11	32	31	+	−
**22**	Green	−	22	26	22	22	+	−
**23**	Green	−	1	1	2	0	−	−
**24**	Green	−	3	5	2	5	−	−

Red – denotes a strong positive Milk ELISA reading. Amber – denotes an inconclusive Milk ELISA reading. Green – denotes a negative Milk ELISA reading.

a Represents the most recent Milk-ELISA status.

b Values show the numbers of plaques obtained in two independently tested samples.
